# Effects of Topographic Variation on Soil Fungal Community Structure in a *Podocarpus oleifolius* D. Don Tree Plantation

**DOI:** 10.3390/biology15090720

**Published:** 2026-05-01

**Authors:** Lina Marcela Anacona-Finscué, Paola Torres-Andrade, Adriana Corrales, Adriana María Marín Velez, Jorge Andres Ramírez

**Affiliations:** 1 Facultad de Ciencias Agrarias, Universidad del Cauca, Popayán 190003, Colombia; linafin@unicauca.edu.co; 2 Society for the Protection of Underground Networks, SPUN, Dover, DE 19901, USA; adriana@spun.earth; 3 Departamento de Planeacion Smurfit Westrock Colombia, Cali 760501, Colombia; chaquiroromeron@gmail.com

**Keywords:** forestry, understudied ecosystems, tropical montane forest

## Abstract

Soil fungal communities play a central role in nutrient cycling and ecosystem functioning in tropical montane forests, yet the relative influence of topographic heterogeneity and soil depth on their assembly remains poorly understood. This study evaluated the composition, diversity, and functional structure of soil fungal communities associated with an experimental *Podocarpus oleifolius* plantation in the Colombian Andes. Using ITS2 rDNA sequencing, fungal assemblages were characterized from soil samples collected around ten trees distributed along a topographic gradient. For each tree, samples were collected at two soil depths (0–10 cm and 10–20 cm), yielding a total of 17 samples after quality control. Topographic variables derived from a digital elevation model were used to evaluate their influence on community structure. A total of 1875 Operational Taxonomic Units (OTUs) were detected, dominated by Ascomycota. No significant differences in alpha or beta diversity were observed between soil depths. In contrast, slope emerged as the strongest environmental driver of community composition. A high proportion of unassigned OTUs highlighted the presence of poorly characterized fungal diversity. These findings highlight the importance of incorporating fine-scale terrain heterogeneity into restoration strategies with native species and into future studies of soil microbial dynamics in tropical montane ecosystems.

## Simple Summary

Soil fungi are tiny organisms that live underground and play a crucial role in maintaining forest health. These organisms help plants absorb nutrients, recycle organic matter, and support the storage of carbon in the soil. However, we still know little about how these fungal communities are organized in tropical mountain forests, especially in plantations of native tree species. In this study, we examined soil fungal communities associated with a *Podocarpus oleifolius* plantation, a native conifer that is considered vulnerable due to habitat loss. We compared fungal communities at two soil depths and across different positions along a slope. We found that changes in the landscape, such as terrain slope, had a stronger influence on fungal communities than differences in soil depth. We also discovered many fungal groups that are still poorly known to science. These results show that small variations in terrain can shape underground biodiversity. Understanding these patterns can help improve forest restoration strategies and support the long-term conservation of native mountain ecosystems.

## 1. Introduction

The functioning of tropical forests depends on the dynamic interaction of biological, geochemical, and physical processes operating across multiple spatial and temporal scales. Among these processes, soil microbiota plays a fundamental role in vegetation establishment and ecosystem functioning by regulating major carbon reservoirs and nutrient fluxes, particularly in nutrient-limited systems [1,2]. Among soil microorganisms, fungi are especially relevant due to their role in organic matter decomposition, pathogen control, and formation of mycorrhizal associations that directly affect plant performance [2,3,4]. In these symbiotic interactions, fungi enhance plant nutrient uptake in exchange for photosynthetically derived carbon [5]. The composition and spatial distribution of soil fungal communities are influenced by environmental factors such as topography, soil depth, and physicochemical properties, which act as ecological filters determining species establishment [3,6,7]. These filters influence biogeographic patterns at broad spatial scales, while processes such as competition and niche differentiation become increasingly important in structuring fungal assemblages at local scales [3,8].

Montane forest ecosystems are well-suited for studying fungal biogeography, as steep altitudinal gradients drive changes in vegetation, climate, and soil properties that shape community structure [9,10]. Tropical montane ecosystems are particularly sensitive to subtle variation along altitudinal and topographic gradients [9]. Previous studies in South American montane forests have documented strong effects of elevation and environmental gradients on fungal diversity and community composition, particularly in Andean ecosystems [11,12,13,14]. However, these studies have primarily focused on broad altitudinal patterns or specific functional groups, with limited attention to the combined effects of topography and soil stratification on fungal community structure.

These gradients influence ecosystem processes such as productivity, nutrient availability, and soil respiration, leading to shifts in soil physicochemical conditions that shape microbial community structure through environmental filtering [9,15]. Relatively small changes in slope, aspect, or landscape position can lead to substantial differences in microclimate, soil moisture, and nutrient redistribution and local availability [16,17]. These fine-scale gradients influence both plant community structure and microbial composition. Fungi drive organic matter decomposition and nutrient mineralization and regulate plant nutrient uptake through mycorrhizal associations; therefore, variation in moisture and soil chemistry along topographic gradients leads to shifts in fungal communities and their effects on plant performance and ecosystem functioning [2]. However, in humid tropical montane forests of the Andes, there is limited understanding of how topographic variation and soil depth interact to structure soil fungal diversity and community composition, particularly in systems dominated by native conifer species.

The Podocarpaceae family comprises conifer trees widely distributed in tropical and subtropical montane forests of the Southern Hemisphere [18,19]. In Colombia, it is represented by the genera *Podocarpus*, *Prumnopitys*, and *Retrophyllum* [20,21]. These species hold local economic value as timber used in construction, carpentry, and small-scale wood products across Andean regions [22]. Beyond their economic importance, Podocarpaceae species play a key ecological role in maintaining the diversity and functioning of tropical montane forests, as their distinctive architecture creates specialized microclimates and contributes to nutrient cycling [23]. They are well adapted to nutrient-poor soils, where their persistence is closely linked to nutrient acquisition strategies mediated by root–fungal interactions. In these environments, they can also influence ecosystem processes by producing recalcitrant litter, which slows decomposition and promotes the retention of nutrients in less-available forms [24]. In recent decades, Podocarpaceae have also gained increasing relevance in ecological restoration programs, particularly in high-elevation Andean ecosystems, due to their adaptation to nutrient-poor soils and their potential contribution to carbon sequestration and ecosystem recovery [25,26]. Among these, *Podocarpus oleifolius* D. Don ex Lambert has a broad geographic distribution ranging from Mexico to northern Peru and Bolivia. In Colombia, *P. oleifolius* occurs in Andean montane forests between 1800 and 3600 m.a.s.l., where it is typically associated with acidic, sandy, nutrient-poor soils [22,27]. In Colombia, *P. oleifolius* is currently classified as Vulnerable (VU) due to significant population declines driven by timber exploitation and habitat degradation [28,29]; whereas at the global scale, it is listed as Least Concern (LC) by the IUCN Red List [30]. Previous morphological and molecular studies have documented the presence of arbuscular mycorrhizal fungi and endophytic associations in species of Podocarpaceae, including members of the genera *Retrophyllum*, *Podocarpus*, and *Prumnopitys* [24,31]. Nevertheless, the role of soil fungal communities in natural forests and plantations dominated by *P. oleifolius* remains insufficiently characterized. There is limited understanding of how soil depth and topographic heterogeneity influence the composition, diversity, and functional structure of these communities in Andean montane ecosystems. This study evaluated the composition, diversity, and functional structure of soil fungal communities in an experimental *P. oleifolius* plantation located in the Colombian Andes. Using ITS2 ribosomal DNA sequencing, fungal communities were characterized at two soil depths (0–10 cm and 10–20 cm) around trees distributed along a topographic gradient. In addition, parameters derived from a digital elevation model were used to assess their influence on community structure. Because plantation systems provide a relatively controlled setting with uniform host identity, they allow clearer isolation of how topography and soil depth influence host-associated fungal communities. We hypothesized that topographic variation would exert a stronger influence on fungal community composition and structure than soil depth. Specifically, we expected that variation in slope and related terrain features would be more strongly associated with shifts in community composition than vertical soil stratification. Thus, the primary objective was to identify the environmental factors shaping soil fungal composition and diversity associated with *P. oleifolius*. Specifically, we addressed the following question: How do fungal community composition and structure vary along a topographic gradient and across soil depths within a *P. oleifolius* plantation? The results contribute to understanding how fine-scale environmental heterogeneity shapes soil fungal communities in tropical montane ecosystems and offer insights relevant to the management of native Podocarpaceae plantations.

## 2. Materials and Methods

### 2.1. Study Site

**Figure 1:**
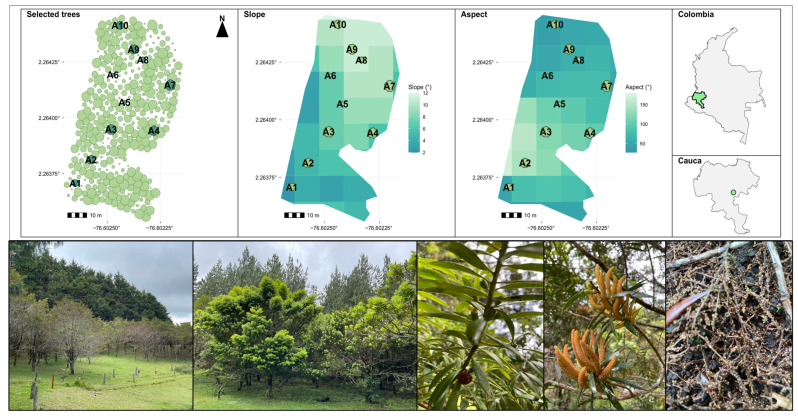
(**Upper panel**) Location of the sampling site, sampled trees (A1–A10), and local topographic variation within the experimental *P. oleifolius* plantation. (**Lower panel**) Representative features of *P. oleifolius*, including stand view, individual trees, reproductive structures (fruits and male flowers), and fine roots.

The study was carried out in a *Podocarpus oleifolius* D. Don ex Lambert experimental plantation located in the municipality of Sotará (Cauca), in southwestern Colombia (2°17′59.4″ N, 76°34′12.8″ W) (Figure 1). The plantation occupies 0.4 hectares and was established in 1998 by the company Smurfit Westrock, Colombia as part of an ecological restoration initiative. Before its establishment, the site had been used for maize (*Zea mays*) and potato (*Solanum tuberosum*) cultivation. Before planting, root cuttings of *P. oleifolius* were inoculated with a spore suspension of the mycorrhizal fungus *Boletus* spp. (5 kg per 500 L of water), obtained from nearby pine plantations. The plantation was established using a 3 × 3 m spacing, and each tree received 60 g of NPK fertilizer (14–35–9) and 15 g of 48% borax at the time of planting. Since its establishment, the plantation has not undergone any further silvicultural interventions, allowing the site to develop under minimal management. The site is located within a lower montane, very humid forest, along an altitudinal gradient from 2400 to 2450 m.a.s.l. The mean annual temperature is 14.5 °C, and annual rainfall is approximately 2043 mm, distributed in a well-defined bimodal pattern. The volcanic soils are moderately to slightly acidic and rich in organic carbon in the upper 100 cm of the soil profile [22].

### 2.2. Selection of Trees and Soil Sampling

The plantation consisted of 347 individuals for each tree; total height and diameter at breast height (DBH) were measured. From this group, ten trees with DBH exceeding 10 cm and good phytosanitary conditions were randomly selected along the topographic gradient for further analysis. Overall, trees had an average basal area of 0.03 m^2^, ranging from 0.02 to 0.07 m^2^, and an average height of 7.45 m, with individual heights between 4.90 and 11.10 m. Soil sampling was conducted in April 2023. Around each of the ten selected trees, two soil samples were collected at 50 cm from the trunk in opposite directions. Each sample set included two depths: surface (0–10 cm) and deeper soil (10–20 cm). Samples used for analysis consisted of soil adhering to fine roots. These samples were divided into two subsamples: one for fungal DNA extraction and the other for soil physicochemical analysis. For the molecular analysis, 0.25 g of soil was placed into sterile 1.5 mL tubes, kept cool during transport, and stored at −20 °C until further processing. For physicochemical analysis, two composite samples (1 kg each) were created by combining soil from all ten trees per depth, and sent to Campolab Ltd. (Yumbo, Colombia). Analyses included pH, organic matter content, total nitrogen, available phosphorus, and cation exchange capacity (Table 1). Because these samples were composite and not replicated, physicochemical measurements were used to characterize the general edaphic conditions of the study site and were not included as explanatory variables in statistical analyses of fungal community structure.

### 2.3. DNA Extraction and ITS Sequencing

Fungal DNA was extracted from 0.25 g of soil using the 2× cetyltrimethylammonium bromide (CTAB) protocol [32]. Before DNA extraction, soil samples (10 trees at 2 depths) were homogenized under sterile conditions using a TissueLyser (Qiagen, Hilden, Germany) with beads. For taxa identification, the internal transcribed spacer 2 (ITS2) region of nuclear ribosomal DNA was selected as the barcode marker. Amplification was carried out using the universal primers ITS3 (5′-GCATCGATGAAGAACGCAGC-3′) and ITS4 (5′-TCCTCCGCTTATTGATATGC-3′) [32]. To assess the quality of the extracted DNA, measurements were taken using a NanoDrop™ 2000 spectrophotometer, (Thermo Fisher Scientific, Waltham, MA, USA). Sequencing (2 × 300 bp, paired-end reads) was then conducted on the Illumina MiSeq platform (Illumina, San Diego, CA, USA) by Novogene Bioinformatics Technology Co., Ltd. (Beijing, China).

### 2.4. Bioinformatic Analysis

Raw sequencing data were processed using LotuS2 v2.25 [33] within R v4.2.1 [34], following a pipeline designed to generate operational taxonomic units (OTUs). The workflow included quality filtering based on Phred scores, dereplication of identical reads, merging of paired-end sequences, and the detection and removal of chimeric reads. These procedures reduced sequencing errors and technical artifacts before clustering. Once filtered, high-quality reads were clustered into OTUs using a 97% similarity threshold, implemented through VSEARCH v2.23.0 [35]. Taxonomic identities were assigned by comparing sequences against the UNITE database (version 9.0) [36], applying a minimum confidence threshold of 80% at the genus level. Analyses were subsequently conducted on a curated phyloseq object containing fungal OTUs clustered at a 97% sequence similarity threshold [37]. Prior to downstream analyses, the dataset was filtered to exclude extremely rare taxa defined as OTUs detected in fewer than two samples and phyla with cumulative prevalence ≤ 10 occurrences across all samples. Unassigned OTUs were retained for diversity and community-level analyses [38]. Finally, functional guilds were assigned using FungalTraits v0.0.3 [39], associating OTUs with genus-level functional guild annotations. OTUs with ambiguous or unassigned guild annotations were excluded, and when multiple guilds were available for a given genus, only genera with a single, unambiguous guild assignment were retained for downstream analyses.

### 2.5. Topographic Variables

Topographic data were derived from a 12.5 m resolution digital elevation model (DEM) generated using ALOS PALSAR imagery and accessed via Earthdata Search (NASA, Greenbelt, MD, USA). DEM processing was performed using the raster package v3.6-24 [40], following established protocols for spatial data analysis [41]. Topographic variables, including slope, aspect, and planform and profile curvature, were calculated from the DEM [42], representing surface curvature perpendicular and parallel to the slope direction and influencing flow convergence and acceleration (Table 2). Overall, the plantation is located on a moderately sloping terrain. Aspect values showed that the site predominantly faces northeast to east, suggesting relatively consistent solar exposure across plots. Planform curvature values were slightly positive on average, indicating a predominantly convex microtopographic form, which may reduce lateral water accumulation. In contrast, profile curvature values indicated a balance between concave and convex vertical surface forms. Specifically, for each sampled tree, topographic variables were calculated within a focal area of 81 m^2^. Prior to ordination and PERMANOVA analyses, topographic variables exhibiting Pearson correlation coefficients greater than 0.65 were excluded to reduce collinearity. These multivariate analyses were conducted using the vegan package v2.7-3 [43] in R v4.2.1 [34].

### 2.6. Statistical Analyses

All statistical analyses were performed on the complete soil fungal community associated with *P. oleifolius* using R v4.2.1 [34], with a significance threshold of *p* < 0.05. To assess sampling effort, a species accumulation curve was generated using Biodiversity R v2.16.1 [44]. Mean OTU relative abundances were computed per depth, and the ten most abundant genera were identified for each soil layer. For multivariate analyses, OTU abundance data were Hellinger-transformed to minimize the influence of dominant taxa and to meet the assumptions of linear ordination [38]. Alpha diversity, including observed OTU richness, Shannon, and Simpson indices, was compared between soil depths using non-parametric Wilcoxon rank-sum tests. Beta diversity differences were tested using PERMANOVA, implemented as permutation-based ANOVA on Bray–Curtis dissimilarity matrices (999 permutations) [38,45]. Distance-based redundancy analysis (db-RDA) was used to assess the influence of environmental variables, including topographic features (slope, aspect, planform, and profile curvature), tree structure (basal area and height), and soil depth, on fungal community composition. The statistical significance of constrained ordination models was tested using permutation-based ANOVA (999 permutations, α = 0.05), and homogeneity of multivariate dispersions among soil depths was evaluated using the betadisper procedure using the vegan package v2.7-3 [43]. Non-metric multidimensional scaling (NMDS) was performed using Bray–Curtis dissimilarity matrices derived from the same Hellinger-transformed OTU abundance data to visualize the distribution of the sample units. Connectivity among fungal communities across individual trees was examined using a presence–absence approach, whereby OTU tables were binarized and aggregated at the tree level. The number of shared OTUs between trees was quantified using pairwise co-occurrence counts, which were visualized as weighted, undirected networks [46].

## 3. Results

### 3.1. Community Composition

After quality filtering, a total of 1,769,428 fungal sequences were obtained from 17 soil samples, clustering into 1875 operational taxonomic units (OTUs). Of these, 1,694,305 reads (95.8%) and 1710 OTUs (91.2%) were assigned at the phylum level. However, taxonomic resolution decreased at lower ranks, with only 407,556 reads (23.0%) and 240 OTUs (12.8%) assigned at the genus level. A total of 75,123 reads, corresponding to 165 OTUs, remained unclassified across all taxonomic levels.

**Figure 2:**
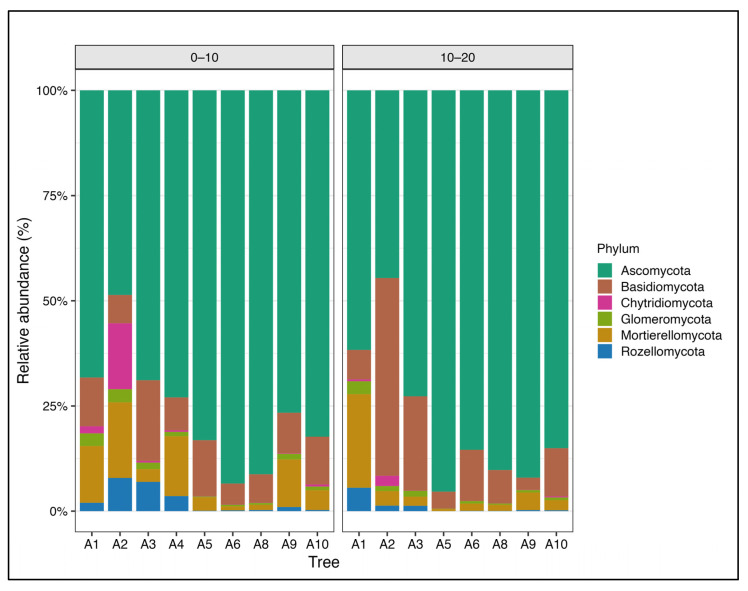
Taxonomic composition of soil fungal communities at two soil depths (0–10 cm and 10–20 cm) in a *P. oleifolius* plantation, showing the relative abundance of dominant fungal phyla based on ITS2 sequence data.

The fungal community was predominantly composed of Ascomycota (75.8%), followed by Basidiomycota (11.6%), Mortierellomycotina (5.1%), and Mucoromycotina (4.4%) (Figure 2). A similar pattern was observed in OTU richness, with Ascomycota and Basidiomycota representing 68.7% and 13.8% of the detected OTUs, respectively.

### 3.2. Fungal Community Patterns Across Soil Depths

Fungal communities showed weak vertical differentiation across the soil gradient. The 0–10 cm depth presented slightly greater richness (1844 OTUs) compared to the 10–20 cm depth (1729 OTUs), with a high degree of taxonomic overlap between depths. In total, 87.1% of OTUs were shared across both layers, while 146 OTUs (10.1%) were exclusive to the surface layer and 31 OTUs (2.8%) were found only in deeper soil. Thus, differences between depths were driven mainly by shifts in relative abundance rather than taxonomic turnover (Figure S1). At the phylum level, both layers were dominated by *Ascomycota* and *Basidiomycota*, though their relative proportions varied with depth. Despite a notable portion of OTUs without genus assignment, the most abundant identified genera were *Leohumicola* (7.4%), *Archaeorhizomyces* (6.3%), *Mortierella* (3.6%), and *Hygrocybe* (1.7%). OTUs within *Leohumicola* and *Mortierella* genera were more abundant in the upper layer, whereas *Archaeorhizomyces* and *Hygrocybe* were more prevalent at 10–20 cm depth.

**Figure 3:**
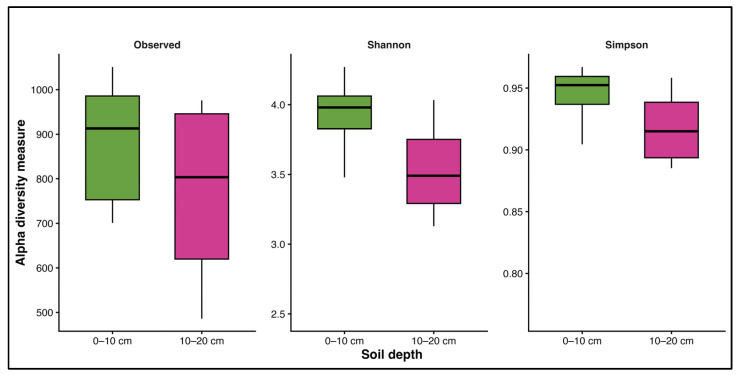
Alpha diversity of fungal communities at two soil depths (0–10 cm and 10–20 cm) in a *P. oleifolius* plantation. Observed OTU richness, Shannon diversity, and Simpson’s diversity (1–D) were calculated from ITS2 sequence data. Sample sizes were *n* = 9 for 0–10 cm and *n* = 8 for 10–20 cm.

Despite these compositional patterns, alpha diversity metrics, including observed OTU richness, Shannon diversity, and Simpson’s index, did not differ significantly between depths (Wilcoxon rank-sum test, *p* = 0.24) (Figure 3). Similarly, beta diversity did not show significant differences between depths (PERMANOVA, *p* = 0.13) (Figure S2).

### 3.3. Drivers of Fungal Community Structure

**Figure 4:**
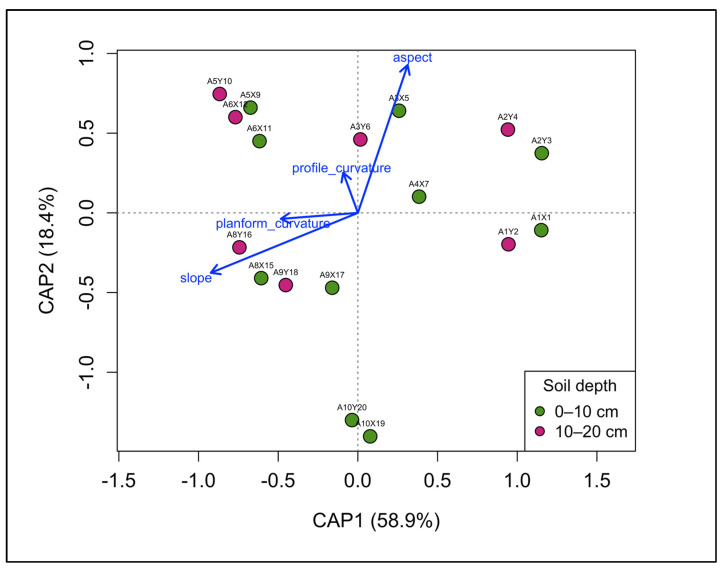
Soil fungal community composition constrained by topography. Distance-based redundancy analysis (db-RDA) ordination of fungal community composition as a function of topographic variables. Points represent individual soil samples and are color-coded according to soil depth (0–10 cm and 10–20 cm). Topographic variables included in the ordination are displayed as vectors, indicating their direction and relative contribution to variation in community composition.

Multivariate analyses indicated that fungal community composition in the *P. oleifolius* plantation was shaped primarily by topographic features. Distance-based redundancy analysis (db-RDA) revealed a significant relationship between community structure and the set of topographic variables—slope, aspect, planform curvature, and profile curvature (Figure 4). Among these, slope was the strongest topographic predictor of fungal community composition (F = 4.93, *p* = 0.001), followed by aspect with a weaker but significant effect (F = 2.10, *p* = 0.035). Planform and profile curvature had no significant influence. The first constrained axis (CAP1) showed the largest explained variance and was strongly associated with slope, whereas the second axis (CAP2) captured additional variation, mainly associated with terrain aspect (Figure 4). Species scores indicated that the effects of topography were distributed across the fungal community rather than driven by a small number of dominant taxa, as no individual OTUs showed strong loadings along the constrained axes. Variation partitioning further confirmed that topographic variables played a much larger role in shaping community composition than tree structural variables or soil depth. Soil physicochemical properties showed limited variation between depths (Table 1), with both layers showing similar texture, particle-size distribution, and acidic pH, consistent with the limited vertical differentiation observed in fungal community composition.

### 3.4. Functional Guilds and Network Connectivity of Soil Fungal Communities

**Figure 5:**
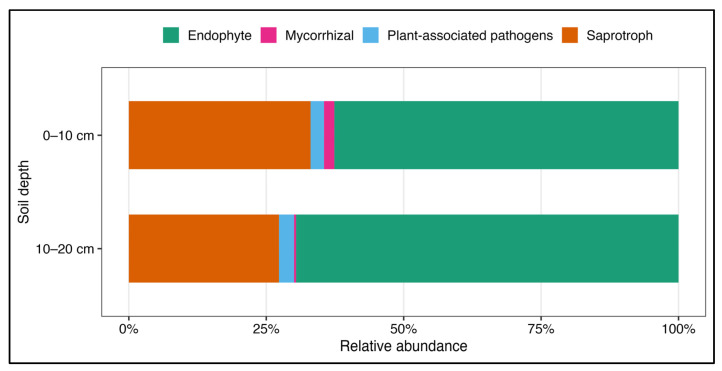
Relative abundance of major fungal functional guilds in soil samples collected at two depths (0–10 cm and 10–20 cm) in a *P. oleifolius* plantation. Guilds were assigned based on genus-level identification and grouped into five major categories. Relative abundances are expressed as percentages of sequence reads.

Overall, only 6.2% of OTUs could be assigned to functional guilds, yet these accounted for 11.9% of total reads (Figure 5). Endophytic fungi dominated both soil depths with multiple OTUs of *Leohumicola*, followed by saprotrophic fungi and plant-associated pathogens. Mycorrhizal OTUs were more abundant in the 0–10 cm depth, whereas deeper soil was characterized by fewer, many of which were taxonomically unassigned (Figure S3). Among mycorrhizal fungi, OTUs of *Ceratobasidium*, *Entoloma*, *Lactarius*, and *Cenococcum* were more abundant in surface soil, while only one OTU of *Russula* was predominant at 10–20 cm. An OTU affiliated with Archaeospora (Glomeromycota), an arbuscular mycorrhizal genus, was detected in our dataset. Plant-associated pathogens showed depth-specific patterns, with *Ganoderma* in the 10–20 cm depth and *Neonectria* concentrated in the 0–10 cm depth. Saprotrophic fungi were mainly represented by *Mortierella* OTUs, with *Blastobotrys* detected only at 10–20 cm. These patterns were consistent with results from indicator species analysis, which showed that only a small fraction of OTUs (3–4%) were significantly associated with soil depth (*p* ≤ 0.05). Most indicator OTUs were associated with the 0–10 cm layer and were primarily assigned to *Mortierella* and *Neonectria*. These belonged mostly to Ascomycota, including orders such as Helotiales, Eurotiales, and Chaetothyriales. In contrast, the 10–20 cm depth harbored fewer indicator OTUs, also mostly within Ascomycota, but many remained taxonomically unclassified.

**Figure 6:**
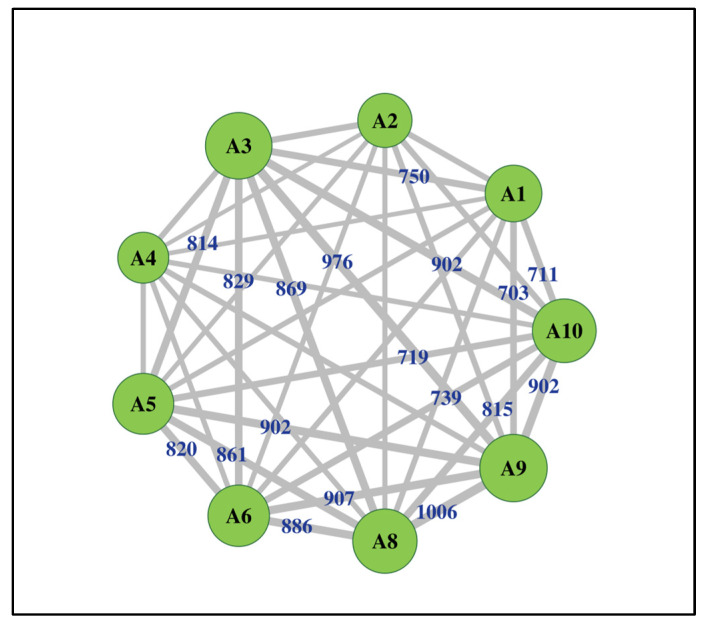
Network representation of shared fungal OTUs among trees in a *P. oleifolius* plantation. Nodes represent individual sampled trees, and edges indicate the number of shared fungal OTUs between tree-associated soil communities. Edge thickness is proportional to the strength of connectivity.

Distance-based redundancy analysis of functional guild composition revealed a significant influence of topography (F = 4.59, *p* = 0.018), driven primarily by the first constrained axis (CAP1; *p* = 0.010) with slope identified as the only significant predictor (*p* = 0.003). An abundance-based indicator analysis further supported depth-related differentiation in functional composition (*p* < 0.05), indicating that distinct functional assemblages dominated different portions of the soil profile. The surface soil (0–10 cm) was characterized by a diverse assemblage of predominantly saprotrophic and fast-growing fungi, whereas deeper soil layers were more strongly associated with taxa commonly linked to roots or long-term persistence in soil. Network analysis indicated a broadly connected fungal community linking all sampled trees through shared OTUs (Figure 6). All individuals were integrated within a single network, although connectivity strength differed among trees. Trees A8, A9, and A10 exhibited higher OTU richness, whereas A2, A4, and A6 showed comparatively lower richness.

## 4. Discussion

The soil fungal community associated with *P. oleifolius* was dominated by Ascomycota (75.8%), followed by Basidiomycota (11.6%) and Mortierellomycota (5.1%). This distribution is consistent with reports from the montane forest and Neotropical systems [4,46], yet taxonomic resolution remained limited. The high proportion of OTUs unassigned at the genus level (87.2%) is a recurring phenomenon in tropical and under-sampled ecosystems, reflecting significant gaps in reference databases and current taxonomic knowledge [4,8,47]. In this context, plantations using native species such as *P. oleifolius* could potentially serve as reservoirs of undocumented fungal diversity and provide an opportunity to further our understanding of ecosystem functioning. These findings highlight the importance of expanding sequencing efforts and taxonomic descriptions in Andean forests.

No statistically significant differences were detected in alpha (*p* = 0.24) or beta diversity (*p* = 0.13) between the 0–10 cm and 10–20 cm soil layers. Associations between fungal diversity and soil physicochemical variables were not evaluated due to the composite nature of the soil samples across depths. This pattern reflects high taxonomic overlap (87.1% shared OTUs); however, inference is constrained by the limited sample size (*n* = 17). Although the number of sampled trees was limited, the sampling design allowed for controlled comparisons across a defined topographic gradient within a relatively homogeneous stand. Nevertheless, increasing the number of sampled trees in future studies would improve the detection of finer-scale spatial variability. Furthermore, vertical stratification was not detectable at the scale of sampling, which may be consistent with the general characteristics of the site [48], including relatively high organic carbon content, potentially limiting the development of strong vertical gradients that structure fungal niches in more strongly stratified soils [49]. Nevertheless, the absence of detectable stratification should be interpreted cautiously, as limited statistical power may have constrained resolution, and finer-scale and deeper sampling could reveal patterns not captured under the present design.

Distance-based redundancy analysis (db-RDA) identified slope as a significant predictor of community composition. Topography was strongly associated with fungal community composition. Rather than functioning as a direct ecological filter, slope likely reflects spatially structured environmental variation [42], potentially related to hydrological processes and the redistribution of soil resources [50]. Thus, increasing slope may influence drainage patterns and runoff, potentially affecting soil water availability, aeration, and lateral nutrient fluxes [42,51]—key constraints on hyphal growth, spore germination, and root colonization. In parallel, steeper or more convex positions are often associated with greater erosion and thinner soils, while downslope or convergent positions tend to accumulate finer particles and organic matter [52], potentially contributing to gradients in texture and fertility that regulate plant nutrient limitation and the associated costs and benefits of mycorrhizal symbiosis [53]. Although the relationship between terrain steepness and fungal assembly was statistically strong within the sampled gradient, clarifying the underlying mechanisms will require finer-scale measurements of water redistribution and microclimatic variation along the slope continuum. Similar topography-driven differences in soil nutrient availability and forest structure have been reported in Andean montane ecosystems, where valley and ridge positions exhibit pronounced contrasts in productivity and species diversity [10,54]. Together, these findings suggest that terrain-mediated heterogeneity may consistently shape both above- and belowground communities in tropical mountain forests [52]. In relation to the central research question, fungal communities varied primarily along the topographic gradient rather than across soil depths, highlighting the dominant role of microtopographic heterogeneity in structuring belowground assemblages in this Andean plantation.

The detection of native ectomycorrhizal (ECM) and arbuscular mycorrhizal (AM) taxa, including Archaeospora, raises the possibility that *P. oleifolius* may engage in multiple mycorrhizal associations to facilitate nutrient acquisition in phosphorus-limited Andean soils. Arbuscular colonization of gymnosperm roots in nutrient-poor montane systems has been previously documented [31,55], suggesting that such associations extend beyond angiosperm-dominated tropical forests. However, DNA detection in soil does not demonstrate active root colonization, so it is possible that the presence of ECM fungi in our dataset could reflect spore dispersal from nearby pine plantations or native oak forests. Confirmation of the symbiotic status of *P. oleifolius* will require direct assessment of root tissues using transcriptomic analyses to verify active colonization and metabolic activity.

A major limitation of the functional analysis is that only 6.2% of OTUs were confidently assigned to functional guilds. The genus *Archaeorhizomyces*, frequently associated with root-influenced soil microhabitats in forest ecosystems [56], was detected within the assemblage, although its functional role in this site remains unclear. Therefore, functional interpretations should be considered with caution, as they are restricted to a small and taxonomically resolved subset of the community and may underrepresent rare or poorly characterized taxa with specialized roles. Co-occurrence network analysis revealed extensive sharing of OTUs among trees, indicating a structurally integrated fungal assemblage across the 0.4 ha plantation. Such patterns are consistent with spatial continuity at the stand scale, but they do not demonstrate physical hyphal connections or resource transfer through common mycelial networks. Experimental approaches, including stable isotope tracing, are necessary to determine whether these co-occurrence patterns correspond to the functional redistribution of nutrients.

## 5. Conclusions

Fungal community structure and composition associated with *P. oleifolius* were most strongly associated with topographic variation, particularly slope, which explained a high proportion of the variance in community assembly. The lack of statistically detectable differentiation between the 0–10 cm and 10–20 cm layers suggests that, within the upper 20 cm of these soils, microtopographic variation may have a stronger influence than vertical gradients. The predominance of Ascomycota and the frequent occurrence of taxa such as *Archaeorhizomyces* and *Leohumicola* were consistent with acidic, organic-rich soils at the site. Network analyses revealed widespread sharing of OTUs among trees, indicating structural connectivity across the site, although functional resource exchange remains to be experimentally verified. Together, these findings highlight the importance of incorporating fine-scale terrain heterogeneity into forest restoration planning and long-term evaluation of belowground community dynamics in high-elevation Andean ecosystems.

## Figures and Tables

**Table 1:** Soil physicochemical properties associated with the experimental *P. oleifolius* plantation at two soil depths.

Variable	0–10 cm	10–20 cm
Texture	Sandy loam	Sandy loam
Sand (%)	67.20	65.70
Silt (%)	18.60	18.60
Clay (%)	14.20	15.70
Base saturation (%)	82.20	88.60
pH	4.78	4.86
Electrical conductivity (dS m^−1^)	0.18	0.29
Organic carbon (%)	5.58	6.74
Exchangeable acidity (meq 100 g^−1^)	0.32	0.48
Potassium (meq 100 g^−1^)	0.13	0.26
Magnesium (meq 100 g^−1^)	0.32	0.76
Calcium (meq 100 g^−1^)	0.89	2.63
Sodium (meq 100 g^−1^)	0.09	0.08
Cation exchange capacity (meq 100 g^−1^)	1.74	4.21
Phosphorus (mg kg^−1^)	3.71	8.12
Sulfur (mg kg^−1^)	3.65	23.20
Boron (mg kg^−1^)	0.29	0.30
Iron (mg kg^−1^)	38.80	43.30
Manganese (mg kg^−1^)	4.97	16.20
Copper (mg kg^−1^)	2.06	1.93
Zinc (mg kg^−1^)	0.81	1.52

Note: dS m^−1^ = decisiemens per meter; meq 100 g^−1^ = milliequivalents per 100 g of soil; mg kg^−1^ = milligrams of element per kilogram of soil. Base saturation was calculated as the sum of exchangeable K, Mg, Ca, and Na divided by cation exchange capacity (CEC) × 100.

**Table 2:** Topographic variables associated with the experimental *P. oleifolius* plantation.

Variable	Mean	Minimum	Maximum	Standard Deviation
Slope (°)	8.76	4.19	12.69	3.28
Aspect (°)	84.41	44.08	109.41	20.74
Planform curvature (×10^3^) *	1.12	−0.06	3.19	1.18
Profile curvature (×10^3^) *	−0.24	−3.91	8.83	3.72

* Dimensionless variables.

## Data Availability

The data supporting the findings of this study are available from the corresponding author upon request.

## Supplementary Materials

The following supporting information can be downloaded at: https://www.mdpi.com/article/10.3390/biology15090720/s1. Figure S1. Taxonomic composition of soil fungal communities at two soil depths (0–10 cm and 10–20 cm) in a *P. oleifolius* plantation; Figure S2. Non-metric multidimensional scaling (NMDS) ordination of fungal community composition across two soil depths (0–10 cm and 10–20 cm) in a *P. oleifolius* plantation; Figure S3. Relative abundance of selected fungal operational taxonomic units (OTUs) grouped by functional guild across two soil depths (0–10 cm and 10–20 cm) in a *P. oleifolius* plantation.
