# Supplementary figures and images for: Effects of Topographic Variation on Soil Fungal Community Structure in a Podocarpus oleifolius D. Don Tree Plantation
Source: Biology (Basel). 2026 May 1;15(9):720. doi: 10.3390/biology15090720 (PMC13162947; doi:10.3390/biology15090720)

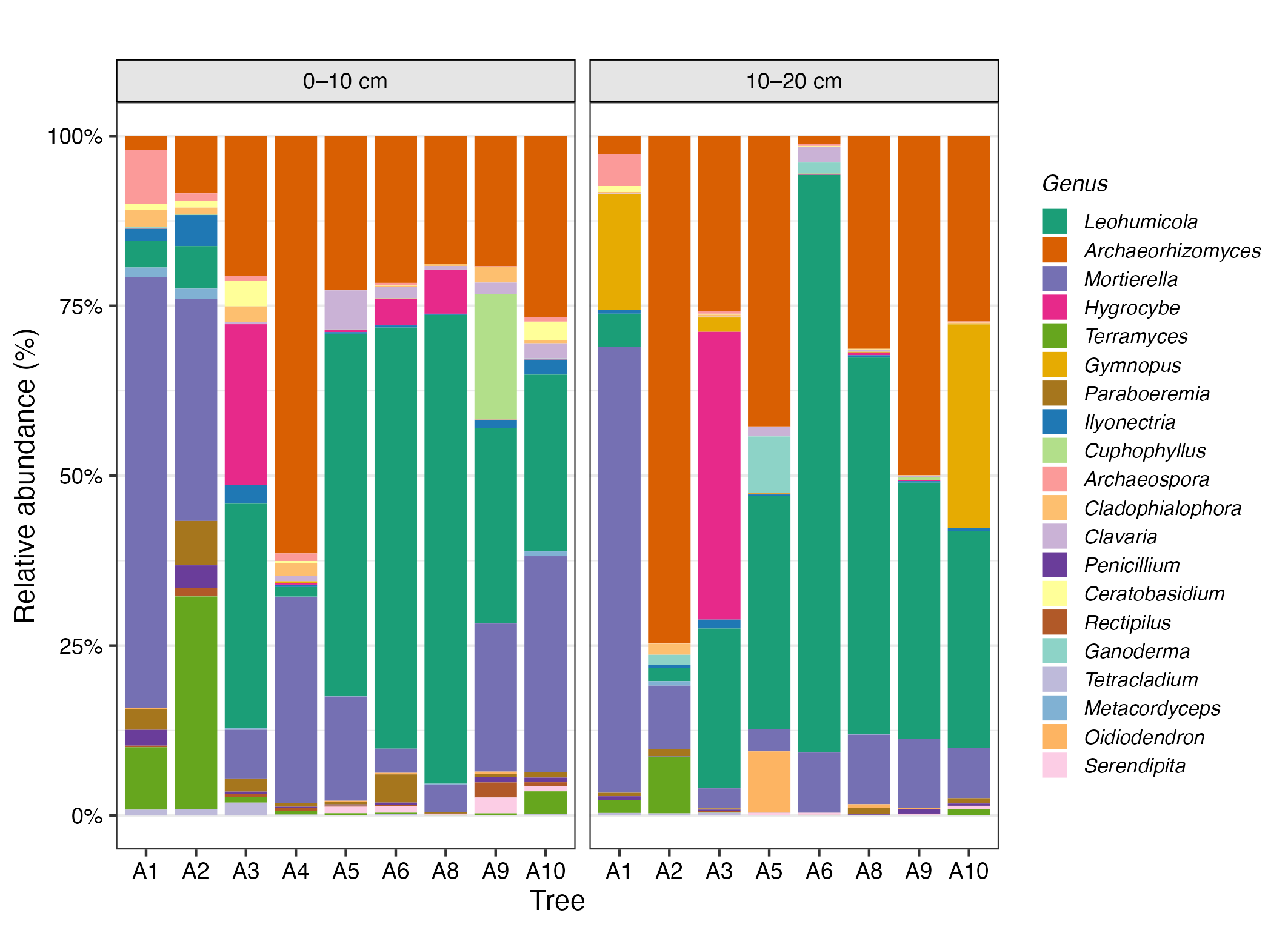

Supplement: Supplementary file 1 [file biology-15-00720-s001.zip › FigS1_20_most_abundant_genera_relative_abundance.tiff]

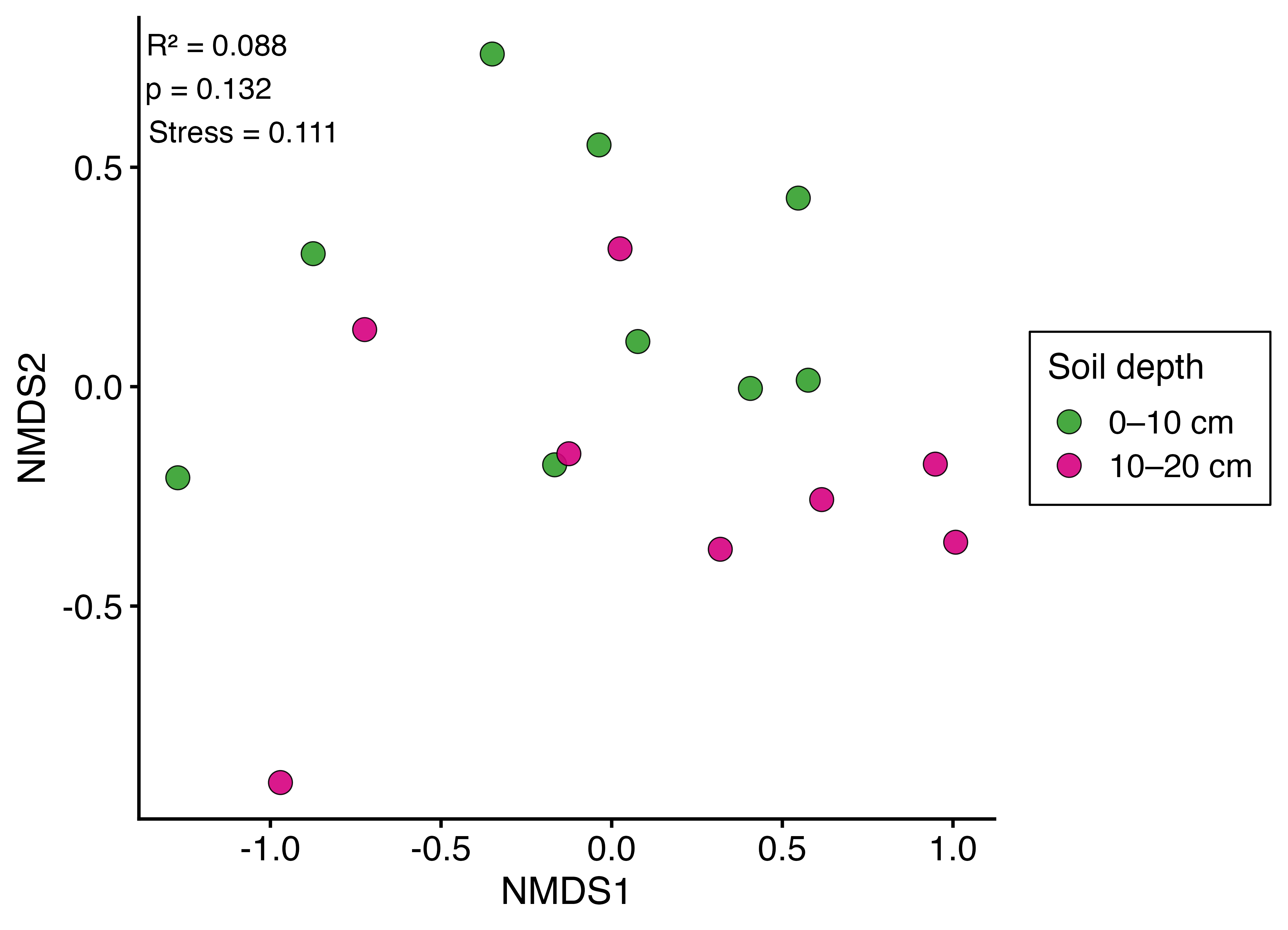

Supplement: Supplementary file 1 [file biology-15-00720-s001.zip › FigS2_Non-metric_multidimensional_scaling.png]

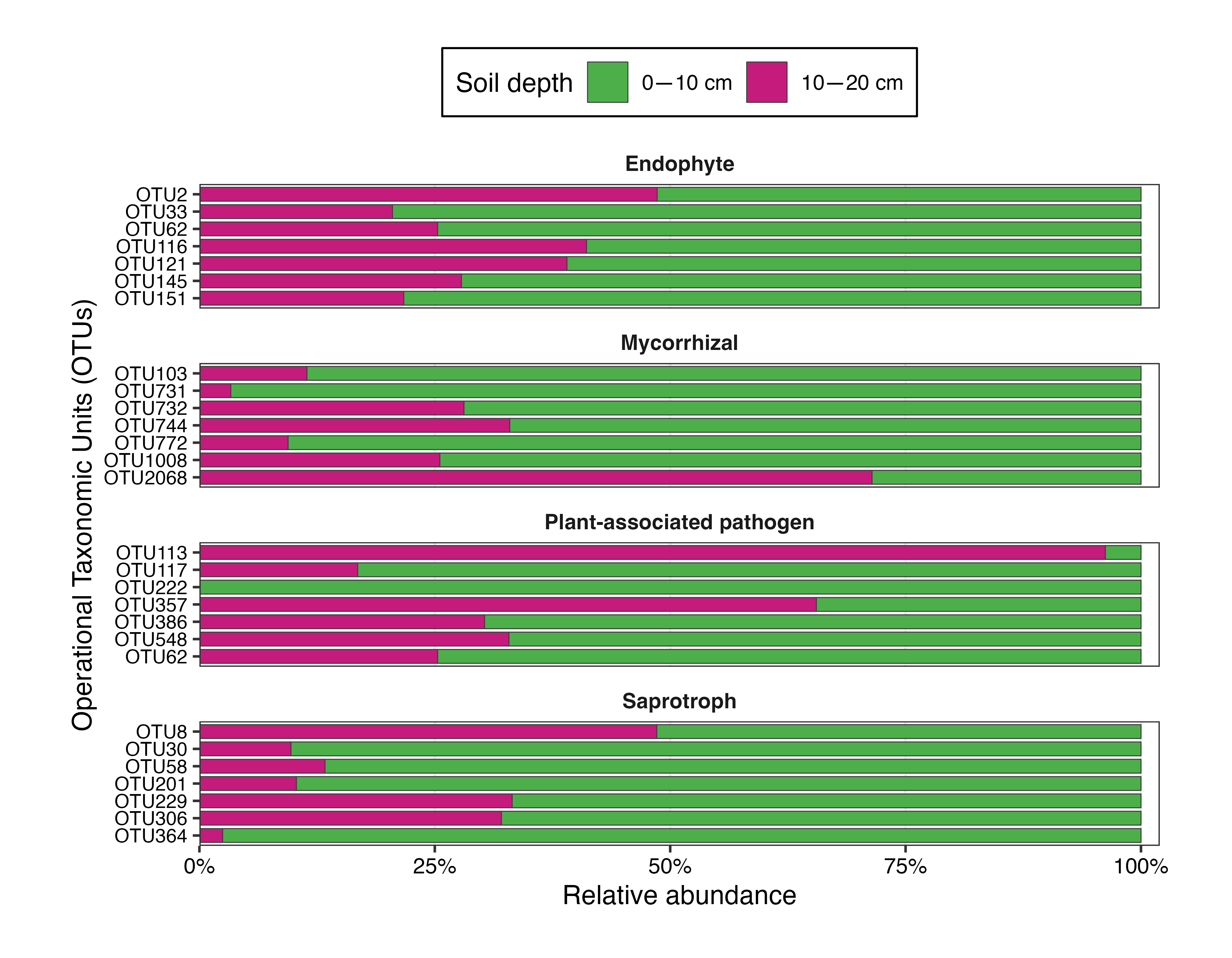

Supplement: Supplementary file 1 [file biology-15-00720-s001.zip › FigS3_Top10_OTUs_by_Functional_Guild_and_Depth.png]
